# “What Doesn’t Kill You Makes You Stronger”: Future Applications of Amyloid Aggregates in Biomedicine

**DOI:** 10.3390/molecules25225245

**Published:** 2020-11-11

**Authors:** Sherin Abdelrahman, Mawadda Alghrably, Joanna Izabela Lachowicz, Abdul-Hamid Emwas, Charlotte A. E. Hauser, Mariusz Jaremko

**Affiliations:** 1 Laboratory for Nanomedicine, Division of Biological and Environmental Science and Engineering, King Abdullah University of Science and Technology, Thuwal 23955-6900, Saudi Arabia; sherin.abdelrahman@kaust.edu.sa; 2 Division of Biological and Environmental Sciences and Engineering (BESE), King Abdullah University of Science and Technology (KAUST), Thuwal 23955-6900, Saudi Arabia; mawadda.alghrably@kaust.edu.sa; 3 Department of Medical Sciences and Public Health, University of Cagliari, Policlinico Universitario, I-09042 Monserrato, Italy; 4 Core Labs, King Abdullah University of Science and Technology (KAUST), Thuwal 23955-6900, Saudi Arabia; abdelhamid.emwas@kaust.edu.sa

**Keywords:** amyloid, aggregation, metals, bioimaging, antiviral, cell penetrating peptides

## Abstract

Amyloid proteins are linked to the pathogenesis of several diseases including Alzheimer’s disease, but at the same time a range of functional amyloids are physiologically important in humans. Although the disease pathogenies have been associated with protein aggregation, the mechanisms and factors that lead to protein aggregation are not completely understood. Paradoxically, unique characteristics of amyloids provide new opportunities for engineering innovative materials with biomedical applications. In this review, we discuss not only outstanding advances in biomedical applications of amyloid peptides, but also the mechanism of amyloid aggregation, factors affecting the process, and core sequences driving the aggregation. We aim with this review to provide a useful manual for those who engineer amyloids for innovative medicine solutions.

## 1. Introduction

Misfolding and successive aggregation of peptides and proteins is a biological process that has been associated with different tissue pathologies [1,2,3,4]. Around approximately 50 peptides and proteins are known to form amyloid fibrils in humans and are associated with diseases [5,6,7]. These include α-synuclein (α-Syn) in Parkinson’s disease (PD) [8,9,10], amyloid beta (Aβ) and Tau in Alzheimer’s disease (AD) [11,12,13], prion protein (PrP) in prion disease [14], and islet amyloid peptide (IAPP), also known as amylin, in type 2 diabetes mellitus (T2DM) [15,16,17,18,19].

The transfer of soluble proteins into insoluble amyloid fibrils requires the creation of intra and intermolecular interactions. At higher concentrations, the stable amyloid state is favored. Therefore, when comparing amyloid structures with their native conformations, fundamental feature differences can be found, which give them unique properties. These include the high level of thermodynamic and kinetic stability under physiological conditions [20,21,22].

The aggregation process is considered to be a cooperative process, which occurs when protein (or peptide) concentrations exceed a critical value [23]. Critical concentration values can be affected by the chemical structure of the biomolecule itself, and by environmental factors such as temperature, pH, and high metal ion concentrations [16,24,25,26]. Many different forms of aggregates can be formed, from amorphous to highly structured aggregates such as amyloid fibrils [27]. Aggregates can originate due to either a non-covalent association of polypeptide chains, or covalent linkage of chains. Recently, Iadanza et al. [7] described extensively mechanisms of fibril and plaque formation, while Chun Ke et al. [2] presented latest advances in atomic structures of amyloid fibrils. Aggregation seems to be a very sophisticated multistep process, therefore, some new attempts of creating a feasible model describing this biological phenomenon have been undertaken in recent years [28,29,30,31]. Particularly of interest, Gillam and MacPhee who described the kinetics of fibril assembly by different models. In addition to the major mechanistic pathways that have been extensively described by Ghosh and De [32]. The authors used five mechanisms to elucidate aggregation processes including (1) aggregation via self-association of monomeric protein, (2) aggregation through conformationally altered protein, (3) nucleation-dependent mechanism, (4) nucleation-independent mechanism, and (5) aggregation through direct chemical linkages and chemical degradation [32].

Recently, interest has grown around biomedical applications of amyloids and amyloid-like peptides [33,34,35,36,37]. The unique features of the amyloid peptide structure enable them to be utilized in versatile biomedical, biotechnological, and nanotechnological applications, etc. [37,38]. The use of amyloid peptides as antiviral agents [39], cell penetrating peptides [40], and their use in bioimaging [41] are among several other interesting applications that require more thorough investigation.

Amyloid plaques were originally thought to be caused by a defect in the amyloid synthesis pathway. However, more recently, the focus has turned towards deficient amyloid beta clearance as the cause. Many reports have correlated defects in the clearance mechanism with the onset of amyloid plaques, rather than overproduction of amyloid beta as was previously suggested [42,43].

Here, we review the amyloid aggregation mechanism, and the factors that affect this process, while cellular regulation and mechanisms of functional amyloid formation were extensively discussed elsewhere [44,45,46]. We also highlight metal ion interactions and their effect on the aggregation process. Selected biomedical applications of amyloid peptides and the clearance mechanisms of amyloid beta are also discussed.

## 2. Amyloid Core Peptides as Model Systems to Study Amyloidogenesis

Around two decades ago, David Eisenberg and his group at UCLA demonstrated that short sequences in functional amyloids, called core sequences, that condensed to small peptide structures of four to seven amino acids, are able to form cross-beta fibrils. Peptide sequences of this short size were identified in amyloid proteins such as amyloid β, tau, amylin, insulin, and Sup35. All short peptides assembled to fibrils and additionally formed microcrystals. Interestingly, both ordered states of the peptides, the fibrillar and the crystal state, resembled each other when their atomic resolution structure was determined [47,48,49]. In search of the chemical clues determining amyloid formation, molecular dynamics simulation studies showed that peptides NFGAIL and NFGAILSS, containing six or eight amino acids, were ideal examples for the study of amyloidogenic sequences assembling to amyloid fibers [50]. These peptide sequences NFGAIL and NFGAILSS are part of the islet amyloid polypeptide (IAPP), effectively forming in vitro fibrillary deposits resembling the in vivo formed fibrils of the whole polypeptide IAPP. Interestingly, NFGAIL hexapeptide fibrils as well as FGAIL pentapeptide fibrils showed similar cytotoxicity to β-pancreatic cells as fibrils from the whole IAPP polypeptide [51]. Thus, sequences of a few amino acids within natural amyloid proteins have been detected and proved to be sufficient as simplified structures for the study of amyloidogenesis. They are now considered as the hotspots within a protein responsible for amyloid formation. The beauty of using short amyloidogenic sequences as model systems lies not only in the ease of synthesizing short peptides, but also in the simplified model to study key residues that are responsible for amyloid formation. These core sequences or hotspots would also ease the search for suitable inhibitor compounds that specifically interact with the amyloid assembly process by inhibiting or slowing down amyloid fibril formation. Multiple investigative studies have searched for the shortest amyloidogenic motif. Analyzing the role of each amino acid residue in short amyloidogenic peptides of IAPP by alanine scanning mutagenesis, Azriel and Gazit proposed a key role of the phenylalanine residue in amyloid formation [52]. Based on their experimental data, they hypothesized that π-π interactions between aromatic residues, in particular phenylalanine, are important for intermolecular recognition and for the assembly towards amyloid fibrils. Further work of Gazit et al. proposed the diphenylalanine dipeptide motif as the shortest amyloid forming peptide [36]. Hauser et al. identified by rational design short aliphatic peptides, coined ultrashort peptides, with three to seven amino acids that are able to form amyloid fibrils [53]. This was the first evidence that aromatic residues might not be as important and crucial as proposed. Further work of the Hauser group demonstrated that these rationally designed aliphatic ultrashort peptides had the same assembly mechanism as short peptides from naturally derived amyloid core sequences such as NFGAIL or other short amyloidogenic peptide sequences [54]. Regarding the amyloid assembly mechanism, amyloid formation is closely dependent on the capability of the involved proteins to take more than one conformation. This is how the name chameleon proteins came into place [29]. We found it intriguing that short to ultrashort amyloidogenic peptides are following the amyloid protein assembly mechanism in a stepwise assembly process from unstructured random coils to structured α-helical intermediate structures ending up in cross-β structures [53]. It is proposed that amyloid aggregates arise by a more general assembly process, which include conformational peptide or protein changes in a stepwise manner [55]. We found that the transition from random coil to an intermediate α-helical conformation is the most crucial step of the process. According to several studies, peptide-membrane or peptide-solvent interactions are considered as the cause for the occurrence of α-helical intermediates. Our own data pointed to the need of a critical concentration of the amyloid peptides in order to form α-helical intermediates [53,54]. To our understanding, α-helical intermediates are necessary for arranging fibrils or nanostructures into stable β-turn fibers. Further work needs to determine if the stepwise assembly process can support the development of novel strategies to control or prevent aggregate formation, in the case of pathogenic amyloids, or to stimulate the formation of the beneficial amyloid type.

## 3. Factors Influencing Aggregation

The pH is one of the most common parameters that significantly influences the physical stability of peptides [56,57], leading to either accelerating or slowing down the rate of aggregation. For instance, as the pH deviates from the protein isoelectric point, the net charge of this protein will increase [58]. The change of pH alters the charge distribution on the molecule’s surface. Proteins/peptides containing histidine residues are highly prone to such changes. An increase of charge leads to the reduction of the aggregation of the protein in aqueous solution due to the enhancement of the electrostatic repulsion between protein molecules that are more charged [58,59]. Interestingly, aggregation can also be enhanced with a pH far away from the protein isoelectric point, by unfolding the structure of the protein. Hence, pH can have opposed effects on the aggregation of a protein [60]. Rui Li et al. [61] used bovine serum albumin (BSA) as a model protein to study the pH effects on protein aggregation and structure. The authors observed an unfolded structural conformation of BSA and an increase in the molecular size, as well as the relative content of the β-sheet upon the gradual decrease of pH from 7.0 to 3.0. The authors concluded that the unfolded BSA structure facilitates formation of insoluble aggregates in the solution and increases protein instability.

Protein aggregation is a temperature-dependent process. The physiological temperature in human cells is crucial to the optimal stability of proteins, thus, even a small alteration of temperature might lead to protein unfolding and nonspecific aggregation [62]. This process can be accelerated or slowed by either cooling or heating the solution [25,27,63]. This will lead to populating/depopulating unfolded or partially unfolded monomer states, which are key intermediates in the aggregation process [63]. Raising the temperature increases the molecular collision rate as well as the hydrophobic interaction frequency, thus accelerating the aggregation rate of the proteins [64]. The lag time of the aggregation at low temperature might be reduced (or even eliminated) at high temperatures, e.g., human IFN-γ aggregation at 1 μM [65]. The temperature change may also alter the secondary structure, which can also alter the aggregation behavior [66].

Additionally, several studies have shown that pressure can affect aggregation rates [67,68,69]. One of the valuable tools for studying protein folding is the high hydrostatic pressure (HHP). This tool has been used to study many proteins that have been associated with amyloid diseases [70,71,72,73]. HHP moves the conformational equilibria to a smaller-volume system, and acts on cavities that are excluded from the solvent (can be found in the native folded proteins’ hydrophobic cores) and aggregates into fibrils [74,75].

Functional amyloids can be rationally engineered into amyloid-based complexes and/or composites through programmed incorporation of non-amyloid materials, thereby bringing new properties into amyloid structures [76]. Among different non-amyloid materials, metal ions are particularly known to promote amyloidogenesis and add new electrochemical and spectroscopical properties to amyloid materials. Moreover, metal binding by amyloids significantly influence their structure and morphology.

When designing and fabricating synthetic amyloid peptides, we need to keep in mind the associated toxicity. Notably, short oligomers (e.g., early stage soluble Aβ aggregates) were suggested to be more closely correlated with neurodegeneration etiology, and may be more closely associated with the severity of pathologies than insoluble, mature fibril deposits [77]. For instance, higher levels of soluble oligomers exist in AD brains compared to healthy ones [78,79]. Metal ions may regulate the toxicity related to amyloidal oligomers by modulating the aggregation kinetics and oligomer conformation. Moreover, metals can also direct the aggregation towards more toxic species, and generate reactive oxygen species [15,80].

## 4. Metal Binding Sites in Amyloid Oligomers and Their Polymerization “Switching” Character

Intensive studies on neurodegeneration processes and possible metal ion participation in the pathological mechanisms have produced numerous data on metal/amyloid interactions (Table 1). Metal coordination studies in vitro and in vivo have often contradictory results. At the base of these discrepancies are different conditions of pH, temperature, ionic strength, and different species concentrations. Moreover, short fragments and/or single domains of the peptide are informative but not fully representative of the whole peptide, especially for depicting the aggregation mechanisms. Nevertheless, we can still benefit from the numerous data of metal/amyloid interactions to build up functional amyloids with versatile applications. The data we gathered in Table 1 can be used in infinite combinations to obtain desired metal/amyloid composite materials. Here, we describe only some peculiar aspects of metal coordination by amyloids.

The amyloid-β peptide (Aβ) is one of the most studied peptides involved in neurodegeneration. Numerous experiments implicated transition metal ions, including Zn(II), Cu(II), and Fe(III), in fibrillar assembly of Aβ and the neuropathology of Alzheimer’s disease (AD) [103]. It was proven that Aβ (1–40) is aggregating into a variety of structures under slightly different assembly conditions [104], and metals clearly contribute to this shifting morphological landscape.

The Zn(II) and Cu(II) metal binding motif of Aβ has been localized to the *N*-terminus 1–28 amino acid fragment [83,105,106,107,108,109,110]. This region also contains pentapeptides, such as KLVFF (Lys-Leu-Val-Phe-Phe) and LVFFA (Leu-Val- Phe-Phe-Ala), which are essential for peptide assembly. These short peptides even alone can self-assemble in vitro into typical amyloid fibrils that are morphologically similar to the full-length Aβ peptide [111], and are nowadays widely used as binding elements to design inhibitors of fibrillation [112].

Independent studies have shown that intermolecular Zn(II) binding can promote Aβ aggregation [108,113,114]. It was reported that Zn(II) ions accelerate the assembly by coordinating two imidazole side chains from different peptide molecules. Different metal coordination structures lead to distinct self-assembled morphologies, ranging from typical amyloid fibrils to twisted ribbons and homogeneous nanotubes [115].

Studies with copper(II) ions and Aβ peptide produced different results. In the longer Aβ(1–40) peptide, an intermolecular His residue-bridging binding site of Cu(II) in the amyloid fibril [108], similar to the Zn(II)-bridged His coordination, was reported [108,111,113,114], whereas other results showed an intramolecular Cu(II)–Aβ complex existing in both soluble and fibrillar Aβ (1– 40) [116]. As both inhibitory and fibril-inducing activities have been reported for Cu(II) [106,117], it is likely that the observed differences in Cu(II) coordination might be directly responsible for the discrepancies in kinetics and morphologies. Furthermore, these different Cu(II)–Aβ coordination structures can be accessed under slightly different experimental conditions.

In order to better understand the assembly differences upon Cu(II) and Zn(II) binding to Aβ, Dong et al. prepared a series of homogeneous Aβ(13–21) and mutant Aβ(13–21)K16A complexes with Zn(II) [111] ions. Their studies confirmed that Zn(II) greatly accelerates the self-assembly rate and induces either typical amyloid fibrils or twisted ribbons and nanotubes depending on metal/peptide stoichiometry [111]. These different morphologies arise from either intra- or inter-sheet His–Zn(II)–His complexation for the fiber and ribbons/nanotubes, respectively [111].

Dong et al. reported that Cu(II) forms complexes with Aβ(13–21) and its K16A mutant, and that the complexes, which do not self-assemble into fibrils, have structures similar to those found for the human prion protein (PrP). *N*-terminal acetylation and H14A substitution, Ac-Aβ(13–21)H14A, alters metal coordination, allowing Cu(II) to accelerate assembly into neurotoxic fibrils. The results show that Cu(II) rapidly disaggregates amyloid fibrils formed by Aβ(13–21)K16A, but is not able to dissociate peptide assemblies formed in the presence of equimolar Zn(II). In contrast to Zn(II) [111], Cu(II) inhibits the assembly of Aβ(13–21)K16A by deprotonating a backbone amide nitrogen and rearranging the peptide backbone to create a chelated metal complex. Although the absent or present metal-ion fibrils displayed similar structures, their morphologies were different.

Generally, the *N*-terminal region of Aβ can access different metal-ion-coordination environments, and different complexes can lead to profound changes in Aβ self-assembly kinetics, morphology, and toxicity. In the absence of metal ions, typical amyloid fibrils and tightly twisted fibers were apparent by atomic force microscopy and transmission electron microscopy (TEM) with diameters of 8 nm. The fibrils formed in the presence of Zn(II) or Cu(II) were both non-twisted and smooth, with diameters of 8–9 nm. It is not only essential metal ions that can bind to Aβ. The interaction of nanoparticles with proteins can affect both protein structure and function, i.e., they can inhibit or facilitate amyloid formation. Wu et al. [118] observed that titanium oxide nanoparticles, widely used in sun creams [119], promote Aβ peptide amyloid aggregation, and act as catalysts for amyloid assembly.

Different amyloid peptides linked to neurodegeneration have metal-binding regions located in the structurally flexible *N*-terminal domain and lie outside the amyloid-determining core [120]. The *N*-terminus of PrP contains four copies of the Cu(II)-binding octa-repeat sequence, PHGGGWGQ [121]. In the presence of two or more molar equivalents of Cu(II), the metal ions bind in an intra-repeat manner, with one histidine imidazole, two deprotonated amides from the next two glycines, and the amide carbonyl of the second glycine as metal binding sites [122]. It is also possible that at low Cu(II) concentrations or under acidic pH conditions, multiple His imidazoles form different octa-repeats bind copper ions and form intramolecular, intermolecular, or combined inter-repeat binding sites [123,124].

Superoxide dismutase 1 (SOD1) is a Cu-Zn metalloprotein that catalyzes the dismutation of the superoxide anion, and its disfunction is correlated with the progressive neurodegenerative disease, amyotrophic lateral sclerosis (ALS). Cu(II) and Zn(II) bind and organize a loop region of SOD1, and mutations related to the metal binding site lead to misfolding and aggregation of SOD1 [125,126]. The oxidative modification and aggregation of SOD1 has also been associated with AD and PD [127]. Moreover, it was speculated that AD, PD, and ALS share common mechanisms of metal ion regulation and/or metal-ion induction of protein aggregation, which might lead to an analogue pathogenic mechanism [111].

α-Synuclein (α-Syn), a 140 amino acid presynaptic protein, is the main component of the fibrillar Lewy bodies in dopaminergic neurons of Parkinson’s disease patients. α-Synuclein (α-Syn) is intrinsically unfolded, and Cu(II) binding leads to rapid aggregation [128]. Different experimental studies have suggested that oligomeric forms of α-Syn constitute the major neurotoxic species, nevertheless such oligomers have never been isolated in vitro or in living cells. Recently [129], a prepared and characterized low molecular weight covalently bound to oligomeric species of α-Syn. Such species were obtained by cross linking via tyrosyl radicals generated by blue-light photosensitization of the metal complex with ruthenium(II)tris-bipyridine in the presence of ammonium persulfate. Compared to the unmodified α-Syn monomer, the photoinduced covalent oligomeric species demonstrated increased toxic effects on differentiated neuronal-like cells.

Gold complexes may inhibit the aggregation of amyloid peptides mainly by metal coordination [95,130]. Some Au complexes showed good inhibitory effects on amyloid peptides, such as PrP106–126, hIAPP, and Aβ protein [94,131,132,133]. Recently, innovative gold complexes, dichloropyrrolidine dithiocarbamate Au complex and dichloro-4-4′-dimethyl-2,2′-bipyridyl Au(III) chloride, effectively inhibited the amylin fragment hIAPP19–37 peptide aggregation and scattered the aggregates into nanoscale particles [95]. These gold complexes bind to the peptide mainly through metal coordination, hydrophobic interaction, and electrostatic interaction. Moreover, the Au complexes exhibited remarkable effects on the regulation of the peptide-induced cytotoxicity and reduction of oligomer formation.

## 5. Functional Metal/Amyloid Complexes

Bacterial biofilms are new tools for bionanomaterial fabrication and can be engineered for different applications under bacteria growth-controlled conditions. Seker et al. [134] engineered E. coli biofilms for use as conductive biopolymers to interface with electrodes and connect bacterial populations to electronic gadgets. Three repeats of aromatic amino acids were added to fiber-forming peptide sequences to produce delocalized π-clouds such as those observed in conductive polymers. In this way, the nonconductive *E. coli* biofilm was switched into a conductive form by genetically inserted conductive peptide motifs containing different combinations of tyrosine and tryptophan. Moreover, the biofilm constructed with CsgA proteins was fused with various gold-binding peptides. Gold nanoparticles were anchored onto the modified CsgA fibers upon the addition of HAuCl_4_ and sodium citrate. With a gold enhancement process on the mineralized CsgA fibers, the manufactured bio-hybrid materials exhibited good conductivity.

Amyloid fibrils are ultra-stable even across a wide range of temperatures, and can be formed from short peptides of low cost [135]. For these reasons, amyloids can be used as effective scaffolds for enzyme immobilization for recyclable catalysis [136]. Moreover, certain short amyloidogenic peptide sequences, in the presence of metal ions, can be utilized as building blocks for catalytic reactions, i.e., acyl ester hydrolysis [137]. Rufo et al. [138] used the zinc complex with a 7-amino-acid peptide to catalyze the hydrolysis of *p*-nitrophenylacetate. Catalytic amyloids have also been explored to catalyze different reactions, including the reversible conversion of CO_2_ to bicarbonate [139], and the hydrolysis of ATP to ADP and AMP [140].

Amyloids derived from food proteins are a new frontier in advanced materials exploitation in biomedicine, tissue engineering, environmental science, nanotechnology, and material science, as well as in food science. Food-derived amyloids have outstanding stiffness and a broad availability of functional groups on their surfaces [141] without exhibiting toxicity. Moreover, some food-derived proteins fibrilize in the presence of transition metals and heating at low pH, e.g., β-lactoglobulin fibrilizes in the presence of Cu(II) and Fe(III) ions [142,143]. Thermal denaturation leads to the exposure of hydrophobic residues, which increases hydrophobic attraction that overcomes electrostatic repulsion, and triggers the aggregation of amorphous aggregates. Hill et al. [144] found that net attraction causes precipitation, while interaction of repulsive charges causes amyloid formation. Moreover, thermal protein unfolding leads to the exposition of amino acid side chains with metal binding ability.

Unlike functional amyloids, amyloids derived from denatured proteins usually cannot be directly utilized as functional molecular materials on their own. However, they can be coupled with other nonamyloid materials to form complex assemblies or composite materials (“nanocomposites”) with new functionalities. Recently, biodegradable amyloid fibrils of β-lactoglobulin (BLG), economic milk protein with natural reducing effects, were proposed as efficient carriers for iron fortification in anemia pathology. Cysteine residues in BLG contain sulfhydryl groups, which are redox active and can be easily oxidized and form disulfide bonds, normally contributing to protein tertiary structures. During fibrillization, BLG unfolds making cysteine residues more accessible in the bulk, thus enhancing the reducing effect. Indeed, mixing FeCl_3_ solution with BLG fibrils leads to the reduction of Fe(III) into Fe(II) ions. Feeding the rats with these hybrid materials did not result in abnormal iron accumulation in any organs, or changes in whole blood glutathione concentrations, inferring their primary safety.

## 6. Biomedical Applications of Amyloid Peptides

Due to their unique structural properties, amyloid peptides are known to have numerous applications in tissue engineering and biomedicine [37,38,145]. In this section, we mainly focus on the antiviral properties of amyloid peptides, and their use in combination with cell penetrating peptides for several applications and in bioimaging. Those applications are among the most interesting and least studied but have great potential if employed efficiently.

### 6.1. Antiviral Activity of Amyloid Peptides against Human Viral Infections

Viruses are one of the main causative factors for serious human diseases that in some instances can be fatal. The process of developing efficient vaccines against viruses is time consuming and costly [146,147], which makes the use of proper antiviral drugs essential in times of pandemics such as the current situation with COVID-19. Few antiviral drugs are approved and available on the market, and those mainly target four groups of viruses: Human immunodeficiency virus (HIV), herpes, hepatitis, and influenza viruses [148,149,150]. Antimicrobial peptides (AMPs) are short, positively charged peptides that are among the first line of defense of the immune system. The most studied AMPs in humans and other mammals are histatins, cathelicidins LL-37, and defensins [151,152,153]. These peptides usually kill pathogens using channel-forming mechanisms, which rely on the formation of ion channels in planar lipid bilayers and cellular membranes. This leads to cytotoxicity and calcium dysregulation of cells [154,155]. Many of these AMPs can form β-sheets, similar to that of amyloid peptides, and β-sheet-forming peptides of a suitable length can form channels such as AMPs. These reports and others indicate a crucial role of the β-sheet structure of those peptides and their role as antimicrobial peptides [156,157,158]. Although a range of amyloid AMPs with antibacterial activity has been reported, only a limited number with antiviral properties have been identified so far [159]. Antimicrobial peptides with antiviral properties are referred to as antiviral peptides (AVPs) in many reports [160,161,162,163]. The balance between peptide net charge and hydrophobicity was found to be an important factor that determines the efficacy of the peptide in many therapeutic applications. For AMPs, the correlation between these factors and the efficiency of peptides against bacteria was thoroughly studied [164,165,166], but this has not been the case for AVPs. However, generally, AVPs have a positive net charge, and are cationic and amphipathic in nature. Data suggests that hydrophobicity plays an important role in the antiviral activity of peptides against enveloped viruses [167,168,169]. The efficacy of AVPs was studied in several viral diseases, including hepatitis [170], herpes simplex [171,172], HIV [173,174], and influenza [175,176]. The mode of action of AVPs is generally one of three: Virus-cell membrane fusion and virus attachment inhibition, viral envelope disruption, or inhibition of viral replication, which, for the influenza virus, involves interaction with its polymerase complex subunits [177]. As interest is increasing in this class of peptides, a research group has created an online database for experimentally tested AVPs (AVPdb—http://crdd.osdd.net/servers/avpdb/) containing comprehensive information including peptide sequences, experimental validation, targeted viruses, and physiochemical properties [178,179,180,181]. However, only a few among the studied AVPs were amyloids in nature, despite the reported efficiency of amyloids as AMPs against bacterial infections, which makes it an interesting area of future research. To this end, Eimer et al. showed that amyloid beta peptides may be associated with the innate immune response to brain infections by the herpes virus by interacting with glycoproteins on the viral particles, thereby entrapping them and leading to the formation of amyloid beta fibers and thus aggregation [182]. Another paper discussed the amyloidogenicity of the PB1(6–13) peptide, which is derived from the influenza virus. Their data showed that this peptide forms amyloid-like fibrillar aggregates. PB1(6–13) peptide was found to have an antiviral activity from previous work from the same team [183]. Through their recent work, they found that this peptide interacts as a monomer to the PB1 of the influenza A polymerase complex, leading to a conformational change caused by a change from the alpha helix to a beta structure. This consequently disrupts the interaction between the PB1 and PA subunits of the influenza A virus polymerase complex, and hence inhibits viral replication [184]. In a very recent paper, researchers employed reverse engineering to synthesize two amyloid peptides each with virus specific aggregation-prone regions (APRs) against influenza A and Zika virus (ZIKV). The two peptides were reported to inhibit replication of the intended viruses with high specificity and without cross reactivity [39].

Antiviral amyloid peptides are a very promising class of antiviral drugs that need more thorough and focused studies especially in our current demanding times. As work in this field is still in its early stages, there is room for discovery and applications are right now in high demand.

### 6.2. Cell Penetrating Amyloid Peptides and Their Applications

Cell penetrating peptides (CPPs) are a class of short peptide sequences, generally 5–30 amino acids in length, that are capable of penetrating cell membranes [185]. The first CPP was reported in 1988 when the human immunodeficiency transcription-transactivating protein (Tat) was found to penetrate the cell membrane and translocate into the nucleus [186]. Recently, in an interesting twist, amyloid peptide sequences were conjugated to CPPs to create a complex that combines cell penetration capabilities with amyloid peptide properties [187].

Veloria and Chen et al. introduced in their paper a new class of amyloid hexapeptides coupled with CPP to selectively target and kill cancer cells [187]. The study aimed to create a new class of anti-cancer peptides (ACPs) that harnesses the toxic effect of amyloids while introducing the capability of entering cells via conjugation with a CPP to target a specific population of cells, in this case cancer cells. Two CPP-amyloid peptide conjugates were studied. The sequence for one of the conjugates was AcPHF6R_9,_ derived from the human Tau protein, particularly the microtubule binding region. Computer scanning of apolipoprotein A_1_ was used to predict the second CPP-amyloid conjugate, AcLAV6R_9_. The aggregation ability of both peptide conjugates was tested and they were found to aggregate with a considerable time lag in comparison to the amyloid peptide without CPP, AcPHF6, and AcLAV6, which aggregated instantaneously. The newly designed amyloid-forming hexapeptides conjugated to the cell penetrating R9 peptide were found to be capable of generating toxic amyloid oligomers stable for hours. It is also worth mentioning that AcPHF6R9 was found to have no toxicity against HEK293 cells, while both amyloid-CPP peptides were toxic to HeLa cells and MDA-MB-231, a highly metastatic mesenchymal stem-like (MSL) subtype of triple-negative breast cancer (TNBC). The selective toxicity of amyloid-CPP peptides towards cancerous cells was attributed to its positive charge, which preferentially interacts with the negatively charged membrane of cancer cells [187].

Another interesting study also by Kokotidou et al. was based on a similar idea of designing cell-penetrating amyloid peptides to be utilized as a gene transfer vehicle [40]. Their idea was based on the increased demand of finding or developing biocompatible carriers of nucleic acids that can efficiently form stable complexes. The complexes can enter the cells without being easily degraded and then release those nucleic acids in the desired location to restore gene function. The negative charge of nucleic acids and their susceptibility to degradation by nucleases makes their internalization into cells not possible without a suitable carrier [188,189,190]. Previous reports discussed the use of amyloid peptides for enhancing gene transfer and retroviral infection. However, the cell entry of amyloid peptides was not reported. The studies focused on two rationally designed amyloid peptides, (NH_3_)^+^–KYRSGAITIGY–CONH_2_ and (NH_3_)^+^–KYKGAIIGNIK–CONH_2_ with cell penetration and DNA binding capabilities. To achieve this, the peptides were designed with β-sheet core sequences that allow the peptide to self-assemble into amyloid fibrils. The core sequence design in this work was based on previous studies from the same team where it was found that ultrashort peptide β-sheet core sequences GAITIG and GAIIG can form amyloid fibrils via self-assembly [191,192]. As CPPs usually consist of mainly positively charged amino acids such as lysine and arginine, which facilitate their internalization into cells, this was also taken into consideration while designing the peptides whereby cationic residues were added to give the amyloid peptides the capabilities to enter the cells and bind efficiently to nucleic acids and virion [193,194,195,196,197]. Kokotidou et al. managed to rationally design two amyloid peptides with cell penetration and DNA binding capabilities that could successfully form stable complexes with luciferase expressing plasmid DNA (pGL3-SV40) and lead to efficient transfection and gene expression. Both CPP amyloid peptides exhibited antibacterial activity while being highly tolerable by mammalian cells [40].

Interestingly, in recent work by Henning-Knechtel et al. [198], amyloid-derived CPPs were used to inhibit Aβ amyloidogenesis. Authors of this work aimed to explore this potential of the CPP against amyloids formation of amyloid beta peptide, which is associated with Alzheimer’s disease. The design of a CPP-based amyloid inhibitor included a hydrophobic sequence from the neural cell adhesion molecule-1 (NCAM1 residues 1–19), which is a plasma-associated glycoprotein similar to PrP. This sequence was coupled to a polycationic PrP_23–28_ sequence that was reported to have a high affinity to Aβ and a net charge of −2.7 at physiological pH. The hydrophobic sequence alone was found to be poorly soluble in water and prone to aggregation, whereas the resulting NCAM1-PrP peptide after coupling with the polycationic sequence was found to be water soluble. A second set of amyloid inhibitors was also designed where the PrP23–28 polycationic sequence was replaced by the Aβ16–20 (KLVFF) sequence. The results show that the sequence, which is derived from the amyloid beta peptide (analogous to the PrP sequence), inhibits Aβ aggregation when bound to it [199,200,201,202]. However, a lysine residue was added to the *N*-terminus of the polycationic sequence derived from the β-amyloid peptide to increase its solubility and the net positive charge. The resulting sequence (KKLVFF) led to a net charge of +4 to the NCAM1-Aβ construct; this was higher than the NCAM1 alone and lower than the NCAM1-PrP, which had a net charge of +6. Results from this study showed that both CPP constructs inhibited the formation of Aβ amyloid and Aβ42 oligomer. This inhibitory effect was attributed to the higher stability of the Aβ42 interactions with CPPs in comparison to its self-assembly. Interestingly, both CPP constructs led to the successful recovery of cell viability and inhibition of neurotoxicity in mouse neuroblastoma cells (N2a) treated with varying concentrations of Aβ42. The authors claim to have designed CPPs that are general amyloid inhibitors and can be used to target several amyloid diseases due to its efficient cellular uptake mechanism and ability to inhibit the amyloid self-assembly process and its subsequent cytotoxicity. Additionally, ultrashort self-assembling amyloid peptides were used as nanocarriers capable of penetrating the cell membrane for drug delivery. In an interesting recent study, Ac-LIVAGK-NH_2_ (LK6), Ac-LIVAGD-NH_2_ (LD6), and Ac-IVKNH_2_ (IK3) ultrashort nanofibrous peptides were converted to globular nanoparticles using hydrodynamic focusing and solvent exchange in a microfluidics system. These nanoparticles were encapsulated with curcumin as a model drug then cellular uptake was observed in HeLa cells [203].

### 6.3. Amyloid in Bioimaging: Bioorganic Nanodots

Bioimaging is an integral technique in biomedicine and cell biology with versatile applications including disease diagnosis and cellular studies [204]. Detection of fluorescence emission is the most used method in bioimaging and relies on the use of fluorescent agents such as organic dyes [205,206]. Selection of the appropriate fluorescent agent relies mostly on the intended application, and involves the consideration of factors such as optical properties, biocompatibility, and specificity for targets [41]. In this context, recent reports discussed an interesting feature of amyloid polypeptides, which is an intrinsic blue-green fluorescence in their aggregate state. This observation was reported for several peptides, including disease-associated amyloid beta peptides (1–40) and (1–42), lysozyme, and tau. In a comprehensive study, this feature was used as an in vitro label-free method to quantify protein aggregation in amyloids; a crucial aspect of amyloidogenesis [207]. A number of hypotheses have emerged to explain this phenomenon, some of them were contradictory. Some reports attributed this intrinsic emission to the chemical alteration of aromatic residues of the amyloid beta sequence or dipolar coupling between excited states of those residues in the aggregated fibril state [208,209]. In contrast, other reports claimed that this fluorescence emission is not correlated to the presence of aromatic amino acids in the amyloid sequence and was found in sequences that do not contain any. Instead, it was claimed to be associated with electron delocalization in the β-sheet structure due to hydrogen bonds [207,210,211,212]. A more recent hypothesis suggested that the intrinsic fluorescence property of amyloids is not attributed to the presence of aromatic residues or the delocalization of electrons in correlation with hydrogen bonds. Instead, it was suggested to be related to proton transfer between the C- and N- terminus in the amyloid fibrils. This hypothesis was supported by data showing significant differences in optical properties between fully protonated and deprotonated forms of amyloid fibrils. A significant decrease in fluorescence emission was observed in amyloid fibrils formed at strongly alkaline or acidic pH [213,214,215,216].

Inspired by the described intrinsic fluorescence nature of some amyloids, an interesting new class of visible fluorescence (FL) bioorganic nanodots, self-assembled from short peptides of different composition and origin, was introduced. The majority of these bioinspired nanodots could be related to thermodynamically stable disease or non-disease-associated amyloids considering the similarity in fiber morphology and β-sheet formation, in addition to identical visible fluorescence optical spectra [36,217,218]. The β-sheet structure and optical properties were found to be achievable also in ultrashort, di- and tri- peptide nanostructures [219]. In recent work by N Lapshina et al., non-fluorescence, di- and tri- aromatic and aliphatic peptides nanodots (PNDs) were fabricated [41]. These PNDs were thermally transformed to visible fluorescence PNDs by subjecting them to 160 °C in ethylene glycol, which leads to refolding of the secondary β-sheet structure. Some of the heated peptide nanodots showed tunable optical properties with fluorescence effects that were excitation dependent from deep blue (420 nm) to red (650 nm). This class of new bioinspired fluorescent peptide nanodots are attractive candidates for bioimaging applications from many aspects; having multicolor emission, small nanometer size that can be utilized to specifically image single cells of small regions, photostability and being potentially biocompatible, which makes using them for live imaging applications an interesting option [41].

Another recent report discussed the use of amyloid-like ultrashort peptides as second harmonic generation (SHG) probes. Several amyloid-like ultrashort peptides examined in this study were found to be SHG-active showing strong signals equivalent or higher than that of collagen, and quadratical dependence of SHG intensity to power intensity [220]. Using SHG microscopy to detect amyloidosis was examined in corneal biopsies of lattice corneal dystrophy patients versus biopsies from normal corneas. A distinct difference in the organization of collagen fibers between normal and diseased corneas could be observed using SHG, which suggests that it can be used as a minimal-invasive diagnostic tool. Thus, this feature would make this class of amyloid-like peptides useful tools for label-free bioimaging techniques and diagnostic purposes [221].

## 7. Amyloid Clearance Mechanisms

Amyloid peptides were found to be a useful and attractive tool for several biomedical applications, including as therapeutic agents or carries of drugs and genetic material into cells [36,37,38]. Some of the interesting applications of amyloid peptides are discussed above. However, their numerous biomedical applications might raise concerns about their fate in vivo. Moreover, clearance of amyloid beta peptide has been found to be more strongly correlated to the accumulation of amyloid beta peptide seen in amyloidogenesis rather than amyloid synthesis [43]. This makes studies on the clearance mechanism of amyloids important for understanding the pathogenesis of correlated diseases and finding new therapeutic targets.

Several enzymatic and non-enzymatic clearance mechanisms have been reported for Aβ in the brain. The non-enzymatic pathway is based on flux of intestinal fluid (ISF) into the cerebrospinal fluid (CSF) then the drainage pathway of ISF. This is followed by uptake (phagocytosis) by astrocytes and microglial cells, and transport through the perivascular basement membrane (peripheral sink mechanism). This mechanism is regulated by a series of receptors including P-glycoprotein primarily found on the abluminal side of the cerebral endothelium, low-density lipoprotein receptor-related protein 1 (LRP1), and very low-density lipoprotein receptor (VLDLR) [42,222,223]. Several protease enzymes are involved in the enzymatic clearance mechanism [223]. These include insulin-degrading enzyme (IDE), glutamate carboxypeptidase II (GCPII), neprilysin (NEP), and matrix metalloproteinase (MMP)-9 [223]. Most amyloid beta-degrading enzymes act as endopeptidases, cleaving the amino acid within the Aβ sequence. However, others cleave amino acids from the C-terminus, acting as carboxypeptidases [223,224]. Amyloid beta clearance via autophagy is another mechanism that involves the activity of the proteasome, which generally has an important role in the degradation of misfolded or damaged proteins and protein aggregates. Autophagy usually catabolizes abnormal macromolecules and organelles, and then further degradation of the resulting macromolecules is carried out by the lysosomes [225]. Although it has been recently suggested that autophagy is one of the clearance mechanisms, there is still insufficient information about the effect of autophagy in neurons or microglia, which play a role in amyloid peptide degradation.

Several studies discussed amyloid beta synthesis pathways and its correlation with the development of neurodegenerative diseases [226,227,228]. However, the available data on clearance mechanisms are not conclusive, although many studies now suggest that defects in the clearance of Aβ might be the actual cause of its accumulation in AD and other diseases, rather than the rate of synthesis [229,230,231,232,233]. Thus, finding new therapeutic agents that can more effectively remove amyloid plaques may be possible by identifying suitable targets involved in clearance. Moreover, this would provide better insights into the pathogenesis of related diseases [232]. Furthermore, it also gives us a better understanding of the potential of amyloid peptides for biomedical applications.

## 8. Conclusions

Amyloids can be defined as a ‘Frenemy’, i.e., friend and enemy of the human body. Normally, they have a physiologically important role, while in pathological states they form toxic aggregates that assemble into fibrils. Changes in temperature, ionic strength, pH, or other environmental factors might lead to amyloid protein aggregation and cell death. Nevertheless, amyloid fibrils have intrinsic physio-chemical properties that have effectively been used in biomedical applications and bioengineering. Despite a multitude of benefits of engineered amyloid materials, their potential toxicity is an important limiting factor that needs to be addressed before common market use. Therefore, studies on potential applications of such materials in daily life is still “tickling the dragon’s tale”. Thus, appropriate tests on biocompatibility, biological effectivity, and non-toxicity are mandatory if a market introduction is planned, avoiding any irreversible harmful effects of the amyloid biomaterial.

## Figures and Tables

**Table 1:** Reported metal ions/amyloids interactions with identified binding sites and the condition of their occurrence.

Protein	Peptide	pH	Metal	Binding Sites	Ref
Aβ	(Rat) Aβ^1–28^ monomer	7.5	Cu(II)	Asp1, His6, Glu11, His14	[81]
	Aβ^1–40^/Aβ^1–42^ monomer	5.5–7.5	Cu(II)	His6, His13, His14, Tyr10	[82]
	Aβ^1–16^/Aβ^1–28^ monomer	7.4	Cu(II)	Asp1, His6, His13, His14	[83]
	Aβ^1–16^/Aβ^1–40^	7.4	Cu(II)	His6, Glu11, His13, His14	[84]
	Aβ^3–40/42^	6.3–8	Cu(II)	Ala2, His6, His13, His14	[85]
	Aβ^1–16^ monomer	6.5–7.4	Zn(II)	His6, Glu11, His13, His14	[86]
	Aβ^1–28^ monomer	7.5	Zn(II)	Asp1, His6, Glu11, His14	[81]
	Aβ^1–28^ monomer	7.5	Zn(II)	Asp1, His6, Glu11, His13, His14	[81]
	Aβ^1–40^ Two monomers	7.4	Zn(II)	His13 and His14 of two adjacent A peptides	[87]
	Aβ^1–28^ monomer	5.3–8.0	Fe(III)	No significant binding	[81]
	Aβ^1–16^/Aβ^1–40^	physiological	Fe(II)	Asp1, Glu3, His6, His13, His14	[88]
	Aβ^1–28^ monomer	5.3–8.0	Al(III)	No significant binding	[81]
	Aβ^1–40^/Aβ^1–42^	No data	Al(III)	sequence 1–16 and sequence 20–35	[89]
α-Synuclein (α-S)	α-S^1–140^	6.5	Mn(II), Fe(II), Co(II) and Ni(II)	Asp119, Pro120, Asp121, Asn122, and Glu123	[90]
	α-S^1–140^	7.2–7.4	Cu(II)	amino acids 3–9 and 49–52 (STRONGER); amino acids 20–24 and 39–44 (WEAKER); His50	[91]
Human islet amyloid polypeptide (hIAPP, amylin)	IAPP^14−22^/IAPP^15−22^	7.5	Cu(II)	His18, Ser19, Ser20	[91]
	IAPP^1−19^	6.5	Cu(II)	His13, His31	[92]
	IAPP^1−19^	7.45	Zn(II)	His18	[92,93]
	hIAPP^19−37^	No data	Au(I)	possible coordination between the gold and the histidine residue	[94,95]
	hIAPP^1–37^	7.5	Ru(II)	C-terminal of the hIAPP could be involved in the binding	[96,97]
Tau	Human Tau40 isoform (441 aa), K32 comprising residues (Met)Ser198-Tyr394, K32Δcys with Cys291, and Cys322 replaced by Ala	6.5	Cu(II)	^287^VQSKCGS^293^ and ^310^YKPVDLSKVTSKCGS^324^	[98]
	Tau-410, 2N3R (*n*-tau)	7.4	Fe(II)/Fe(III)	clear iron/tau binding and Fe(II)/Fe(III) redox reaction	[99]
	hTau40 and phosphorylated hTau40	7.4	Fe(II)/Fe(III), Cu(II), Zn(II)	hTau40/metal interactions; phosphorylated hTau40/metal no interactions/weak Zn(II) interactions	[100]
Prion	human PrP^91–231^ in either the oxidized α-form or the reduced β-form; PrP^52−98^	8.0	Cu(II), Ni(II),	Cu(II)/hPrP^91–231^ and Ni(II)/hPrP^91–231^ interactions; low affinity to Zn(II) and Mn(II)	[101]
	Octa-repeat region of *N*-terminal (PHGGGWGQ)	7–8	Cu(II)	Coordination mode {Nimid, nN−}	[102]
	PrP^106–126^	7–8	Cu(II), Zn(II), Mn(II)	Strong coordination of Cu(II) and weak coordination of Zn(II) ions by hydrophobic tail PrP^112−126^; Mn(II) coordinated by the His111, Gly124, and Leu125 residues	[102]
